# Epidemiological and microbiological investigation of a large increase in vibriosis, northern Europe, 2018

**DOI:** 10.2807/1560-7917.ES.2022.27.28.2101088

**Published:** 2022-07-14

**Authors:** Ettore Amato, Maximilian Riess, Daniel Thomas-Lopez, Marius Linkevicius, Tarja Pitkänen, Tomasz Wołkowicz, Jelena Rjabinina, Cecilia Jernberg, Marika Hjertqvist, Emily MacDonald, Jeevan Karloss Antony-Samy, Karsten Dalsgaard Bjerre, Saara Salmenlinna, Kurt Fuursted, Anette Hansen, Umaer Naseer

**Affiliations:** 1 Department of Infection Control and Preparedness, Norwegian Institute of Public Health, Oslo, Norway; 2 Department of Microbiology, Public Health Agency of Sweden, Department of Microbiology, Stockholm, Sweden; 3 Department of Bacteria, Parasites and Fungi, Division of Infectious Disease Preparedness, Statens Serum Institut, Copenhagen, Denmark; 4 Finnish Institute for Health and Welfare, Department of Health Security, Helsinki, Finland; 5 European Programme for Public Health Microbiology Training (EUPHEM), European Centre for Disease Prevention and Control (ECDC), Stockholm, Sweden; 6 Finnish Institute for Health and Welfare, Department of Health Security, Kuopio, Finland; 7 University of Helsinki, Helsinki, Finland; 8 National Institute of Public Health, Warsaw, Poland; 9 Health Board, Department of CD Surveillance and Control, Tallinn, Estonia; 10 Public Health Agency of Sweden, Department of Communicable Disease Control and Health Protection, Stockholm, Sweden; 11 Data Integration and Analysis, Division of Infectious Disease Preparedness, Statens Serum Institut, Copenhagen, Denmark

**Keywords:** vibriosis, seawater, emerging pathogens, heatwaves, global warming

## Abstract

**Background:**

Vibriosis cases in Northern European countries and countries bordering the Baltic Sea increased during heatwaves in 2014 and 2018.

**Aim:**

We describe the epidemiology of vibriosis and the genetic diversity of *Vibrio* spp. isolates from Norway, Sweden, Denmark, Finland, Poland and Estonia in 2018, a year with an exceptionally warm summer.

**Methods:**

In a retrospective study, we analysed demographics, geographical distribution, seasonality, causative species and severity of non-travel-related vibriosis cases in 2018. Data sources included surveillance systems, national laboratory notification databases and/or nationwide surveys to public health microbiology laboratories. Moreover, we performed whole genome sequencing and multilocus sequence typing of available isolates from 2014 to 2018 to map their genetic diversity.

**Results:**

In 2018, we identified 445 non-travel-related vibriosis cases in the study countries, considerably more than the median of 126 cases between 2014 and 2017 (range: 87–272). The main reported mode of transmission was exposure to seawater. We observed a species-specific geographical disparity of vibriosis cases across the Nordic-Baltic region. Severe vibriosis was associated with infections caused by *Vibrio vulnificus* (adjOR: 17.2; 95% CI: 3.3–90.5) or *Vibrio parahaemolyticus* (adjOR: 2.1; 95% CI: 1.0–4.5), age ≥ 65 years (65–79 years: adjOR: 3.9; 95% CI: 1.7–8.7; ≥ 80 years: adjOR: 15.5; 95% CI: 4.4–54.3) or acquiring infections during summer (adjOR: 5.1; 95% CI: 2.4–10.9). Although phylogenetic analysis revealed diversity between *Vibrio* spp. isolates, two *V. vulnificus* clusters were identified.

**Conclusion:**

Shared sentinel surveillance for vibriosis during summer may be valuable to monitor this emerging public health issue.

## Introduction

The habitat of *Vibrio* spp. bacteria is fresh and brackish water with moderate salinity. Non-toxigenic *Vibrio cholerae*, as well as several human pathogenic non-cholera *Vibrio* species, including *Vibrio alginolyticus*, *Vibrio parahaemolyticus* and *Vibrio vulnificus*, cause vibriosis after seawater exposure or consumption of contaminated seafood [1]. Clinical manifestations range from mild gastroenteritis and otitis to wound infections that may lead to severe necrotising fasciitis and septicaemia with a potentially fatal outcome [2-5].

The Baltic Sea region is one of the areas where increasing numbers of cases related to *Vibrio* species causing vibriosis (VCV) have been reported in the last decades [6]. Several studies have shown how the occurrence of heatwaves, which lead to an increase in sea surface temperature, are linked with an increase in the number of reported vibriosis cases [4,7-12]. For instance, the years with an especially warm summer in the Baltic Sea region, 2006, 2010 and particularly 2014 (the warmest year in historical records at the time), were also the years with the largest number of vibriosis cases reported [6,11].

However, there is a notable gap in surveillance data for vibriosis since it is not a notifiable disease in the majority of European countries [1,6]. Therefore, the aim of this multi-country study was to describe the epidemiology of vibriosis cases in countries bordering the North and Baltic Seas area during the exceptionally warm year of 2018 [13,14], in order to investigate the extent of these infections in the study countries, map their genetic diversity, understand the predictors for developing severe vibriosis, and propose recommendations for public health measures.

## Methods

### Study design and case definition

We conducted a retrospective study to analyse the epidemiology of VCV infections reported in 2018 in Norway, Denmark, Sweden, Finland, Poland and Estonia, further referred to as the study countries. In addition, Latvia was contacted but no vibriosis cases had been reported in that country. Available data on vibriosis cases since the last warmest summer (2014) were used to contextualise the number of VCV infections in 2018.

We defined a case of vibriosis as a laboratory-confirmed VCV infection from the study countries; those related to travel outside the study countries were excluded. If more than one sample type was recorded concurrently in the same patient, we included only the sample type that indicated a more severe infection. For few cases (n = 18) where more than one *Vibrio* species was recorded concurrently in the same patient, only the species related to a more severe infection type was included.

### Data source and collection

Each country used different data sources including comprehensive compulsory passive surveillance systems for vibriosis (Sweden, Finland, Poland, and Estonia), national laboratory notification databases (Denmark) or nationwide surveys to public health microbiology laboratories (Norway). More details about the national surveillance systems can be found in Supplementary Table S1.

The reporting criteria varied between countries that had a surveillance system in place in 2018. In Sweden, a confirmed case was defined as an isolation of *Vibrio* spp. other than toxigenic *V. cholerae* O1 or O139. In Finland, a case was defined as (i) *V. cholerae* including non-O1, non-O139 identified in a faecal sample by culture or PCR (or other nucleic acid detection), (ii) *V. parahaemolyticus* identified in a faecal sample by culture or PCR (or other nucleic acid detection) and (iii) any *Vibrio* spp. identified in a blood sample or cerebrospinal fluid by culture or PCR (or other nucleic acid detection). In Poland, a case was defined according to the International Classification of Diseases 10th revision, diagnosis A05.3 for *V. parahaemolyticus*. Estonia considered as a vibriosis case any case meeting the clinical criteria (otitis, wound infection, gastroenteritis, septicaemia) and laboratory criteria (detection of *Vibrio* spp., *V. cholerae* non-O1, non-O139 in a clinical specimen detected by any method). Meanwhile, the criteria for *Vibrio* spp. infections reported from national laboratory notification databases (Denmark) and nationwide laboratory surveys (Norway) were based on detection of *Vibrio* spp. other than toxigenic *V. cholerae* O1 or O139.

We compiled the vibriosis cases from all study countries into a harmonised dataset that included: patients’ sex and age group, year and month of infection, country, European nomenclature of territorial units for statistics 3 (NUTS3) region [15], identified VCV, type of sample and, if known, source of exposure and travel status at the probable time of infection. The severity of an infection was inferred from the sample type: blood/serum (n = 60) and wound swabs (n = 144) were considered as a proxy of severe infections, while skin swabs (n = 28), ear secretion (n = 176), faeces (n = 19), urine (n = 2), nasal swab (n = 1) and other unspecified (n = 15) sample types were considered linked to non-severe infections. Seasons were defined according to the northern hemisphere seasons (spring: March to May; summer: June to August; autumn: September to November; winter: December to February). Population data as per 31 December 2018 were publicly available from national statistics authorities.

### Epidemiological investigation and statistical analysis

We describe the epidemiology of vibriosis cases reported in 2018 in the study countries per country and as total counts. Data presented include the sex ratio, notification rate per 100,000 inhabitants, median age, distribution of cases across age groups, season and identified VCV. Case numbers are presented by country and by region (NUTS3) and month of infection was considered for the investigation of seasonality. Severity of infection is described by age group and month of infection. Association of sex, age group, season and VCV with developing severe vibriosis was further analysed by estimation of crude odds ratios (OR) and 95% confidence intervals (CI) by univariate logistic regression analysis. Adjusted OR (adjOR) with 95% CI were estimated in a multivariate analysis. The binary outcome was severe/non-severe vibriosis.

Data analysis was performed using Stata version 15.0 (2017. Stata Statistical Software: Release 15; StataCorp LP, College Station, United States). Categorical variables were described as proportions with 95% CI and compared using chi-squared test. Continuous variables were described using mean and standard deviation or median and range and compared using t-test or non-parametric Wilcoxon rank-sum test. Trends were assessed using a nonparametric test across ordered groups. Observations with missing values for the variables under comparison were excluded from the respective analysis.

We used an alpha level of 0.05 for all statistical tests. Stata outputs of p values p < 0.000 are reported as p < 0.001.

### Sampling of isolates of *Vibrio* species causing vibriosis, MLST and WGS analyses

We collected available clinical VCV isolates in 2018 from the national public health institutes or regional laboratories and complemented them with available clinical (2014–2017) and environmental seawater (2018) isolates. The collected isolates were subjected to whole genome sequencing (WGS); eighteen isolates from Sweden were not subjected to WGS because of resource prioritisation, these isolates’ combination of *Vibrio* species, region and patient sex were already represented in the dataset. DNA was extracted and sequenced using standard operating procedures and Illumina sequencers. The WGS raw files are available at the European Nucleotide Archive (https://www.ebi.ac.uk/ena) under study project accession number PRJEB43461. Accession numbers of all sequenced isolates are listed in Supplementary Table S2.

Raw WGS reads from each country were analysed together using a common pipeline for species identification, multilocus sequence typing (MLST), and phylogenetic analyses. We used BBmap (version 38.69) to clean the raw reads and FastQC (version 0.11.8) to generate quality reports of samples. In addition, we used Kraken2 (version 2.0.8_beta) to confirm the species and Shovill (1.0.9) to assemble (using SPAdes version 3.13.1) the genomes.

We searched the PubMLST database (https://pubmlst.org/) using Ariba (2.14.4). We assigned a sequence type (ST) to isolates of non-toxigenic *V. cholerae*, *V. parahaemolyticus*, *V. vulnificus* and *V. alginolyticus* according to their respective MLST schemes.

We used Parsnp (v1.2) and a neighbour-joining algorithm to build the phylogenetic trees, and Snp-dists (0.7.0) to calculate the single nucleotide polymorphism (SNP) distance between isolates. A cluster was defined as two or more *Vibrio* spp. isolates within 30 SNPs difference. An in-house pipeline was used for sequence mapping, generation of consensus sequences, alignment calculation and SNP filtering (exclusion distance = 300). We used R package ggtree [16] to visualise the phylogenetic trees generated by the in-house pipeline (https://github.com/folkehelseinstituttet/Vibrio-Project).

## Results

### Descriptive epidemiology of vibriosis cases

**Figure 1:**
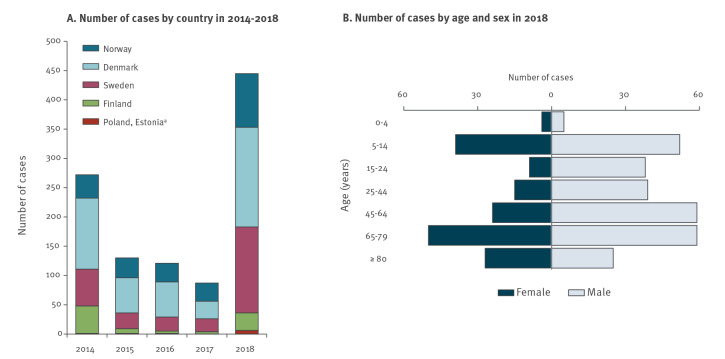
Occurrence of vibriosis cases in study countries during 2014–2018 and distribution of cases by age and sex, northern Europe, 2018 (n = 445)

^a ^Data from Poland and Estonia are reported in an aggregated manner because of the small number of cases.

In 2018, 445 non-travel-related cases of vibriosis were reported in the study countries, which was the highest case number in a single year compared with the four previous years (n = 610) (Figure 1A and Table 1). Additional information on epidemiological parameters of these vibriosis cases per country can be found in Supplementary Table S3.

**Table 1:** Summary of epidemiological parameters of vibriosis cases per species in the study countries, northern Europe, 2018 (n = 445)

	Total vibriosis (n = 445)		*Vibrio* *alginolyticus* (n = 152)		Non-toxigenic *Vibrio cholerae* (n = 100)		*Vibrio parahaemolyticus* (n = 89)		*Vibrio* *vulnificus* (n = 45)		Non-subtyped *Vibrio* spp. (n = 59)	
	n	%	n	%	n	%	n	%	n	%	n	%
Sex												
Female	168	37.8	68	44.7	26	26.0	38	42.7	14	31.1	22	37.3
Male	277	62.2	84	55.3	74	74.0	51	57.3	31	68.9	37	62.7
Age group (years)												
0–4	9	2	2	1.3	4	4.0	0	0.0	1	2.2	2	3.4
5–14	91	20.4	47	30.9	23	23.0	7	7.9	0	0.0	14	23.7
15–24	47	10.6	24	15.8	10	10.0	4	4.5	0	0.0	9	15.3
25–44	54	12.1	26	17.1	12	12.0	7	7.9	1	2.2	8	13.6
45–64	83	18.7	24	15.8	24	24.0	20	22.5	6	13.3	9	15.3
65–79	109	24.5	24	15.8	19	19.0	36	40.5	21	46.7	9	15.3
≥ 80	52	11.7	5	3.3	8	8.0	15	16.9	16	35.6	8	13.6
Season												
Summer	326	73.3	97	63.8	74	74.0	78	87.6	44	97.8	33	55.9
Autumn	96	21.6	45	29.6	22	22.0	6	6.7	1	2.2	22	37.3
Winter	13	2.9	6	3.9	2	2.0	3	3.4	0	0.0	2	3.4
Spring	10	2.2	4	2.6	2	2.0	2	2.3	0	0.0	2	3.4
Country												
Norway	92	20.7	63	41.5	2	2.0	12	13.5	9	20.0	6	10.2
Denmark	170	38.2	70	46.1	3	3.0	55	61.8	16	35.6	26	44.1
Sweden	147	33	19	12.5	64	64.0	19	21.4	19	42.2	26	44.1
Finland	30	6.7	0	0.0	26	26.0	3	3.4	1	2.2	0	0.0
Poland, Estonia^a^	6	1.3	0	0.0	5	5.0	0	0.0	0	0.0	1	1.7
Sample type												
Blood	60	13.5	3	2.0	20	20.0	4	4.5	31	68.9	2	3.4
Faeces	19	4.3	2	1.3	11	11.0	3	3.4	0	0.0	3	5.1
Ear-related	176	39.6	91	59.9	43	43.0	14	15.7	1	2.2	27	45.8
Wound-related	144	32.4	45	29.6	13	13.0	54	60.7	12	26.7	20	33.9
Other	46	10.3	11	7.2	13	13.0	14	15.7	1	2.2	7	11.9
Exposure												
Food/water	6	1.3	2	1.3	3	3.0	1	1.1	0	0.0	0	0.0
Bathing/seawater	109	24.5	17	11.2	38	38.0	12	13.5	25	55.6	17	28.8
Other	1	0.2	1	0.7	0	0.0	0	0.0	0	0.0	0	0.0
Unknown	329	73.9	132	86.8	59	59.0	76	85.4	20	44.4	42	71.2
Severe infection												
Yes	204	45.8	48	31.6	33	33.0	58	65.2	43	95.6	22	37.3
No	241	54.2	104	68.4	67	67.0	31	34.8	2	4.4	37	62.7

^a ^Data from Poland and Estonia are reported in an aggregated manner because of the small number of cases.

The vibriosis notification rates ranged between 0.5 per 100,000 inhabitants in Finland and 2.9 per 100,000 in Denmark. Because of the limited number of cases (n = 6), we did not calculate the notification rate for Poland and Estonia. The majority of the cases were male (n = 277; 62.2%) (Table 1) and the largest number of cases were reported in the age group 65–79 years (n = 109; 24.5%) followed by age groups 5–14 (n = 91; 20.4%) and 45–64 years (n = 83; 18.7%) (Figure 1B, Table 1).

Most of the infections were caused by *V. alginolyticus* (n = 152; 34.2%), followed by non-toxigenic *V. cholerae* (n = 100; 22.5%), *V. parahaemolyticus* (n = 89; 20.0%), *V. vulnificus* (n = 45; 10.1%), and non-subtyped *Vibrio* spp. (n = 59; 13.3%). The most common type of infections reported were ear infections (n = 176; 39.6%), followed by wound infections (n = 144; 32.4%) (Table 1).

We observed a difference in the proportions of species affecting each age group. The proportions of *V. vulnificus* and *V. parahaemolyticus* infections followed an upward trend with increasing age (both p < 0.001), with the opposite pattern for *V. alginolyticus* (p < 0.001), and no trend was observed for non-toxigenic *V. cholerae* infections (p = 0.081) (Table 1).

Information on exposure was systematically collected in two countries (Norway and Sweden) that contributed with 239 cases to this study. The reported exposures from these two countries were seawater/bathing (n = 107; 44.8%), food/water poisoning (n = 6; 2.5%), other (unspecified) (n = 1; 0.4%) or unknown (n = 125; 52.3%). However, because the number of cases with unknown information on exposure from all participating countries was larger (n = 329; 73.9%) than that reported in Norway and Sweden, the overall exposure for seawater/bathing from the study countries resulted to be lower (n = 109; 24.5%) (Table 1).

### Geographical distribution of vibriosis cases

**Figure 2:**
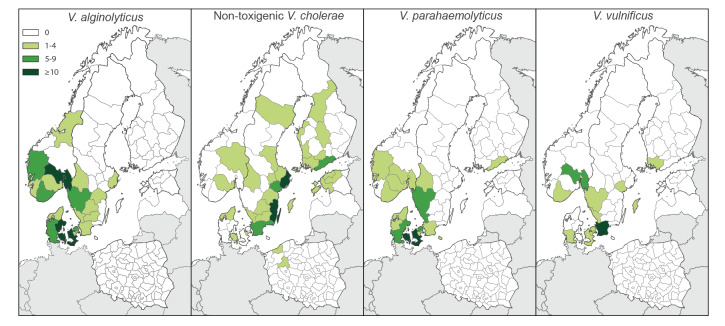
Geographical distribution (NUTS3 level) of vibriosis cases, by identified species, in the study countries, northern Europe, 2018 (n = 445)

The geographical distribution of the vibriosis cases differed between *Vibrio* species (Figure 2).

*Vibrio alginolyticus* and *V. parahaemolyticus* infections were reported mainly from regions adjacent to the North Sea as well as around the connecting sounds between the Baltic and the North Sea: southern and western regions of Norway, all of Denmark and the south-west coast of Sweden (Figure 2A and 2C). Non-toxigenic *V. cholerae* infections were almost exclusively reported from coastal regions of the Baltic Sea: the east coast of Sweden and regions in Finland, Poland and Estonia (Figure 2B). *V. vulnificus* infections, similar to *V. alginolyticus* and *V. parahaemolyticus* infections, mainly occurred in the coastal regions around the connecting sounds between the Baltic and the North Sea, particularly Oslo fjord in Norway, south-west Sweden and Denmark (Figure 2D).

### Severity of infections with *Vibrio* species causing vibriosis 

**Figure 3:**
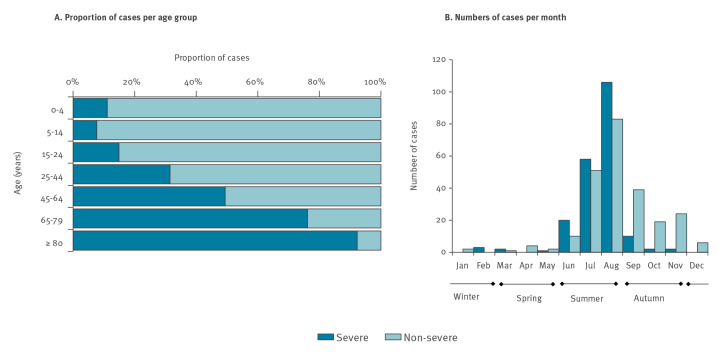
Severity of vibriosis cases in the study countries, northern Europe, 2018 (n = 445)

The proportion of severe VCV infections increased significantly with increasing age (p < 0.001) and it differed by *Vibrio* species (p < 0.001) (Figure 3A and Table 2).

**Table 2:** Predictors without and with adjustment of severe and non-severe vibriosis cases in the study countries, northern Europe, 2018 (n = 445)

Characteristics All cases (n = 445)	Severe infections		Non-severe infections		Univariate logistic regression^a^ OR (95% CI)	Multivariate analysis^a^ adjOR (95% CI)
	n	%	n	%		
	204	45.8	241	54.2		
Sex						
Female	89	53.0	79	47.0	1	1
Male	115	41.5	162	58.5	0.6 (0.43–0.93)	0.7 (0.42–1.27)
Age group (years)						
0–4	1	11.1	8	88.9	0.3 (0.03–2.35)	0.1 (0.01–1.69)
5–14	7	7.7	84	92.3	0.2 (0.07–0.47)	0.1 (0.05–0.41)
15–24	7	14.9	40	85.1	0.4 (0.14–1.02)	0.4 (0.16–1.26)
25–44	17	31.5	37	68.5	1	1
45–64	41	49.4	42	50.6	2.1 (1.04–4.35)	1.9 (0.86–4.18)
65–79	83	76.1	26	23.9	6.9 (3.37–14.33)	3.9 (1.73–8.68)
≥ 80	48	92.3	4	7.7	26.1 (8.1–84.2)	15.5 (4.41–54.31)
Season						
Summer	184	56.4	142	43.6	7.6 (4.13–13.93)	5.1 (2.40–10.86)
Autumn	14	14.6	82	85.4	1	1
Winter	3	23.1	10	76.9	1.8 (0.43–7.19)	3.1 (0.52–18.04)
Spring	3	30.0	7	70.0	2.5 (0.58–10.88)	1.5 (0.27–8.49)
*Vibrio* species						
*V. alginolyticus*	48	31.6	104	68.4	0.9 (0.55–1.61)	1.6 (0.79–3.31)
Non-toxigenic *V. cholerae*	33	33.0	67	67.0	1	1
*V. parahaemolyticus*	58	65.2	31	35.8	3.8 (2.08–6.94)	2.1 (1.00–4.49)
*V. vulnificus*	43	95.6	2	4.4	43.7 (9.96–191)	17.2 (3.28–90.45)
*Vibrio* spp.	22	37.3	37	62.7	1.2 (0.62–2.36)	2.1 (0.86–5.30)

adjOR: adjusted odds ratio; CI: confidence interval; OR: odds ratio.

^a^ Data from Poland and Estonia were not included in the logistic regression analyses.

We observed the highest proportion of severe infections for *V. vulnificus* (95.6%) and *V. parahaemolyticus* (65.2%), while these were lower yet substantial for non-toxigenic *V. cholerae* (33.0%) and *V. alginolyticus* (31.6%) (Table 1 and 2). The exposure for these severe infections with non-toxigenic *V. cholerae* and *V. alginolyticus* was largely unknown (48% and 70%, respectively) or cases were exposed to seawater/bathing (48% and 25%, respectively). In terms of age, these infections were shifted slightly towards the younger age group. In contrast, more than 70% of the severe *V. vulnificus* and *V. parahaemolyticus* infections occurred in the age groups 65–79 and ≥ 80, while 58% and 41% of the severe non-toxigenic *V. cholerae* and *V. alginolyticus* cases, respectively, occurred in these age groups.

The majority of VCV cases occurred in summer (n = 326, 73.3%; ranging per species from 63.8% to 97.8%) (Table 1). No difference in the seasonal distribution of vibriosis cases was observed between countries. According to our multivariate model, the likelihood of developing a severe infection was significantly increased among elderly people (65–79 years: adjOR = 3.9; 95% CI: 1.7–8.7; ≥ 80 years: adjOR = 15.5; 95% CI: 4.4–54.3), for infections caused by *V. vulnificus* (adjOR = 17.2; 95% CI: 3.3–90.5) or *V. parahaemolyticus* (adjOR = 2.1; 95% CI: 1.0–4.5), as well as for infections occurring in summer (adjOR = 5.1; 95% CI: 2.4–10.9) (Table 2).

### Microbiological and molecular investigations

In total, 178 isolates were sequenced in this study. We analysed whole genome sequences of 142 clinical VCV isolates from 2018 that were available at the national public health institutes (non-travel-related n = 135; travel-related n = 7). In addition, we included 23 available clinical VCV isolates from the period 2014 to 2017 (non-travel-related n = 14; travel-related n = 9) as well as 13 Finnish environmental (seawater) non-toxigenic *V. cholerae* isolates from 2018 to investigate the genetic diversity of *Vibrio* spp. in the study countries. More detailed information about the analysed isolates can be found in Supplementary Table S4.

### Phylogenetic analysis

**Figure 4:**
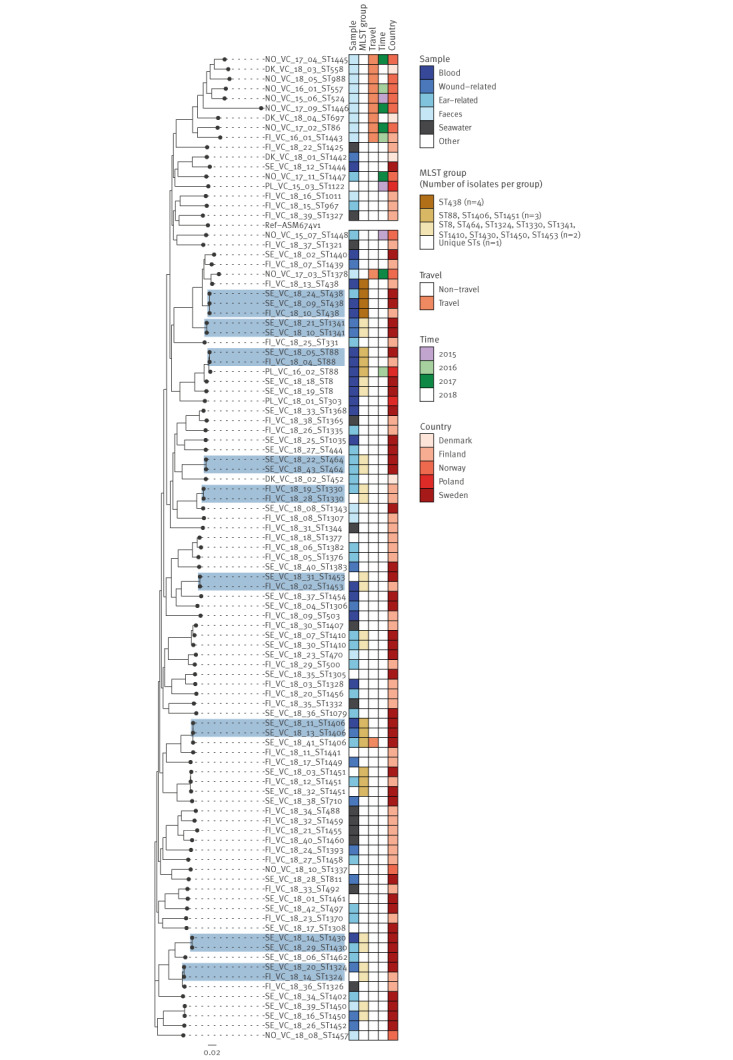
Single nucleotide polymorphism-based phylogeny of non-toxigenic *Vibrio cholerae* genomes from the study countries, northern Europe, 2015–2018 (n = 100)

DK: Denmark; FI: Finland; MLST: multilocus sequence typing; NO: Norway; SE: Sweden; SNP: single nucleotide polymorphism; ST: sequence type; VC: non-toxigenic *V. cholerae*.

The SNP analysis showed a high diversity of isolates for all species. Nine clusters with two or three cases each of non-toxigenic *V. cholerae* isolates were identified in Sweden (n = 4), Sweden/Finland (n = 4) and Finland (n = 1) (Figure 4). Cases whose isolates clustered were sampled within a short time frame (median: 7 days; range: 2–86 days) but detailed information on place of infection was not available.

Nine non-toxigenic *V. cholerae* clusters are shaded in blue. The non-toxigenic *V. cholerae* ASM674v1 sequence was used as reference. The scale bar indicates number of substitutions per site. The first number represents the isolation year and the second number denotes the isolate number.

**Figure 5:**
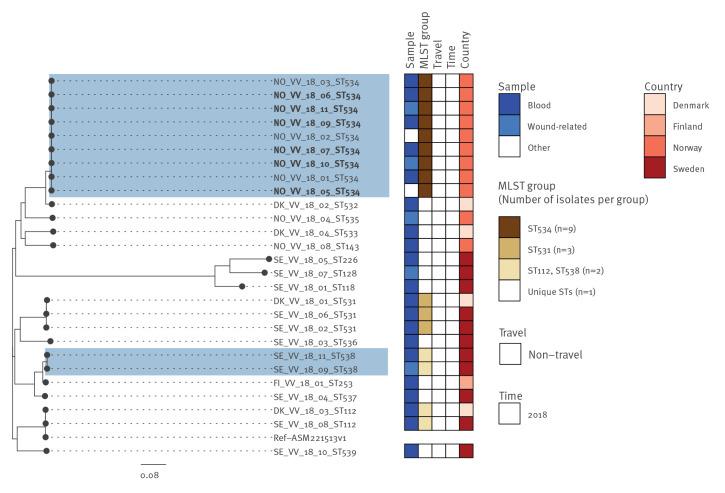
Single nucleotide polymorphism-based phylogeny of *Vibrio vulnificus* genomes from the study countries, northern Europe, 2018 (n = 27)

DK: Denmark; FI: Finland; MLST: multilocus sequence typing; NO: Norway; SNP: single nucleotide polymorphism; SE: Sweden; ST: sequence type; VV: *V. vulnificus.*

In addition, two clusters of *V. vulnificus* isolates with < 10 SNPs difference were detected (Figure 5): one cluster with nine isolates in Norway, where the cases had been infected within 40 days and ca 60 km apart, and one cluster with two isolates in Sweden, where the cases had been infected 30 days and ca 55 km apart.

Two *V. vulnificus* clusters with ≤ 30 SNPs difference are shaded in blue. The *V. vulnificus* ASM221513v1 sequence was used as reference. The scale bar indicates the number of substitutions per site. The first number represents the year of isolation and the second number denotes the isolate number. Six *V. vulnificus* isolates from an outbreak in Norway are shown in bold.

No clusters of *V. alginolyticus* or *V. parahaemolyticus* were identified and more details on phylogenetic analysis for these species can be found in Supplementary Figures S1 and S2.

### MLST analysis

Among the 178 isolates included in this study, 20 groups of isolates had the same ST. Of these, 10 groups were pairs of isolates from a single country (Norway, Sweden or Finland), three were pairs from two countries (Sweden/Denmark n = 1 and Sweden/Finland n = 2), six included three or four isolates each, and the largest group of nine *V. vulnificus* isolates (ST534) was detected in Norway (Figure 4 and 5). Finally, a single *V. parahaemolyticus* isolate from Norway, found in a gastrointestinal infection in spring of 2014, was identified as the pandemic ST3 [17].

## Discussion

Our study provides a detailed overview on the occurrence of vibriosis in the Nordic and Baltic Sea regions in 2018. In a context of epidemiological and microbiological findings as well as studies conducted from 2014 to 2018 [11,12], our results highlight the importance of vibriosis as a concern to public health in this geographical area. Even though the data had been collected using different systems, the study countries reported similar patterns of sex and age group distribution in the affected population. Two-thirds of all vibriosis cases from 2014 to 2018 occurred in the years 2014 and 2018, reported as two remarkably warm years in the literature [6,11-14]. Moreover, although *V. vulnificus* infections are usually considered rare in this region [18], 45 such infections were detected in 2018, while in the preceding years, only eight (2014), none (2015), one (2016) and two (2017) *V. vulnificus* infections were identified. Interestingly, one *V. vulnificus* case occurred at latitude ca 60 degrees north in Finland, which, to the best of our knowledge, is the highest northern latitude at which *V. vulnificus* has been reported. These findings suggest that this pathogen may spread to different areas following seawater warming [19].

It is well documented that vibriosis is more frequently reported in summer [2,6,11,12,19]. Our results from 2018 confirm this pattern, with the majority of infections occurring in summer months. In addition, in this study almost half of all infections reported in 2018 were categorised as severe infections that also mainly occurred during summer season. Mild ear infections may have longer reporting delays up to several months until a patient seeks medical care [20,21] explaining why reporting of mild vibriosis extended to autumn and winter compared with rapidly developing severe blood or wound infections which were mainly reported in summer. However, more accurate information on the probable infection date would be needed to confirm this hypothesis. Few severe infections occurred in autumn, winter and spring, these did not differ from the severe cases that occurred in summer in terms of male/female ratio, reporting country, affected age group or *Vibrio* species. The likely source of infection was available for a subset of cases and suggested that the mode of transmission was mostly through seawater rather than through consumption of contaminated seafood.

The majority of vibriosis cases in the study countries were domestic and males were more frequently affected than females, consistent with other reports [12,22]. Even though the majority of cases were among adults, about a fifth of the detected cases were among children up to 14 years of age, who mostly had ear infections and mild vibriosis; severe infections on the other hand were found to be associated with increasing age. This is probably due to underlying conditions being overrepresented among elderly people. In addition to increasing age, we also found that being infected by *V. vulnificus* or *V. parahaemolyticus* was a risk factor for a more severe VCV infection, probably because these microorganisms are more pathogenic [1,2]. On the other hand, although non-toxigenic *V. cholerae* and *V. alginolyticus* predominantly cause mild infections, in our study, about one third of the cases infected by these species were sampled from blood/serum or wounds. Thus, in absence of systematic data on hospitalisation and symptoms, these infections were also considered as severe. These cases had a lower median age compared with cases infected with *V. vulnificus* and *V. parahaemolyticus*, and the exposure was largely unknown with only some cases exposed to seawater/bathing. Given that a substantial proportion of cases were classified as severe, non-toxigenic *V. cholerae* and *V. alginolyticus* should therefore not be underestimated in vibriosis diagnosis, as has been pointed out previously [23].

We observed geographical disparity in the distribution of VCV in the study countries. *V. alginolyticus* and *V. parahaemolyticus* infections were concentrated in the coastal regions connecting the North Sea to the Baltic Sea, including the Danish sounds, where *V. vulnificus* was mainly reported. Infections with non-toxigenic *V. cholerae* were mostly detected along the coasts of the Baltic Sea. This is in line with previous environmental detection of *Vibrio* species in different areas [2,24-27] and reported clinical *V. vulnificus* infections from Germany [4]. Reasons for the geographical disparity could be related to differences in sea surface temperature and salinity, which represent major factors influencing *Vibrio* spp. growth, and are continuously monitored in the *Vibrio* suitability tool from the European Centre for Disease Prevention and Control [28]. Additional factors, such as phytoplankton composition and nutrient presence in the water [24-26,29], could also have played a role. Additional research studies on the water environment and presence of *Vibrio* spp. in seafood could provide useful information on the ecological niches and geographical distribution of such bacteria, particularly for species associated with a potentially severe clinical outcome.

Our MLST analysis showed a genetic heterogeneity between clinical *Vibrio* spp. isolates, the majority of which belonged to STs not yet assigned not yet assigned in the PubMLST database. The SNP-based phylogenetic analysis revealed small clusters of non-toxigenic *V. cholerae*, containing two to three isolates each, without a clear epidemiological link. That the same non-toxigenic *V. cholerae* strains are detected in one or more countries might be due to common exposure to contaminated seafood or environmental spread of clones through e.g. sea currents [26], plastic pollutants [30], ship ballast water [31] or waterbirds [32].

The occurrence of two *V. vulnificus* clusters, one in Norway and one in Sweden, detected 30–40 days apart and within an area of around 50–60 km, highlights the possibility of emerging *V. vulnificus* clones that caused infections after seawater exposure during the exceptional warm summer in 2018. This was further supported by the epidemiological investigations of the first reported waterborne outbreak caused by *V. vulnificus* after seawater exposure; this outbreak involved six *V. vulnificus* infections from which isolates were included in our study [33]. Moreover, the smaller number of vibriosis cases reported per year during 2019 and 2020, respectively 50 and 52 cases in Norway and 51 and 91 cases in Sweden, further confirms the hypothesis that the risk of vibriosis is higher during warmer summers.

Some limitations apply to our investigation. There were differences in data sources and data availability between the study countries. Notification rates should therefore be compared carefully as vibriosis is not notifiable in all study countries or not for all species. Especially mild infections might have been reported with a delay and/or under-reported. Conversely, in some cases a disease could have been misclassified as vibriosis when the identified *Vibrio* species were merely opportunistic microorganisms present at the site of infection. Case severity classification used in this analysis was not reported directly in any study country, but was inferred based on the sample type. In addition, cases without known travel history were considered as non-travel related, which could have led to an overestimation of vibriosis cases in the Baltic Sea region. Furthermore, the place of residence was used as proxy when place of infection was not available. Regarding the molecular findings, SNP analysis needs to be evaluated carefully since recombination is one of the major sources of genomic changes in *Vibrio* spp. Therefore, the removal of changes caused by recombination could have provided better insight from an evolutionary perspective. Finally, laboratory methodology, capacity and priorities to diagnose and report VCV infections probably differed among the study countries.

During our investigation, we performed a systematic and consistent analysis of epidemiological data from different countries and combined it with the genomic analysis of strains from cases to achieve a comprehensive understanding of the occurrence of VCV infections in this affected region. In addition, following results from two laboratory surveys carried out in Norway and Sweden, we do not have evidence that any factors changed over time and influenced the monitoring in the study countries. All available isolates representing a vibriosis case were included in the study, reducing the risk of bias in the selection procedure. Further source attribution studies, based on epidemiological and/or genomic data, could provide additional information on the burden of vibriosis in relation to possible different source of infections per *Vibrio* species.

Despite the low incidence, severe VCV infections are clinically costly [34], and predictions of changing climate as well as population and socioeconomic projections for the upcoming years suggest that they are likely to increase in the future when growth conditions become more favourable for VCV [19,35].

## Conclusion

It is of interest to detect and report the VCV infections in countries bordering the Baltic Sea and connecting regions to the North Sea to further monitor the situation, especially during summer heatwaves. Moreover, such surveillance would facilitate risk assessments and allow for targeted interventions, including risk communication to raise awareness among clinicians and populations at risk of vibriosis. Countries without comprehensive surveillance could benefit from establishing or expanding dedicated surveillance systems to detect and prevent vibriosis cases. In particular, a shared sentinel system during summer months might be highly valuable.

## Supplementary Data

591000 Supplement

## References

[1] Baker-Austin C Oliver JD Alam M Ali A Waldor MK Qadri F Vibrio spp. infections. Nat Rev Dis Primers. 2018;4(1):8. 10.1038/s41572-018-0005-8 30002421

[2] Collin B Rehnstam-Holm AS . Occurrence and potential pathogenesis of Vibrio cholerae, Vibrio parahaemolyticus and Vibrio vulnificus on the South Coast of Sweden. FEMS Microbiol Ecol. 2011;78(2):306-13. 10.1111/j.1574-6941.2011.01157.x 21692819

[3] Kuhnt-Lenz K Krengel S Fetscher S Heer-Sonderhoff A Solbach W . Sepsis with bullous necrotizing skin lesions due to vibrio vulnificus acquired through recreational activities in the Baltic Sea. Eur J Clin Microbiol Infect Dis. 2004;23(1):49-52. 10.1007/s10096-003-1056-6 14655036

[4] Frank C Littman M Alpers K Hallauer J . Vibrio vulnificus wound infections after contact with the Baltic Sea, Germany. Euro Surveill. 2006;11(8):E060817.1. 16966781 10.2807/esw.11.33.03024-en

[5] Schirmeister F Dieckmann R Bechlars S Bier N Faruque SM Strauch E . Genetic and phenotypic analysis of Vibrio cholerae non-O1, non-O139 isolated from German and Austrian patients. Eur J Clin Microbiol Infect Dis. 2014;33(5):767-78. 10.1007/s10096-013-2011-9 24213848 PMC3996285

[6] Semenza JC Trinanes J Lohr W Sudre B Löfdahl M Martinez-Urtaza J Environmental suitability of Vibrio infections in a warming climate: an early warning system. Environ Health Perspect. 2017;125(10):107004. 10.1289/EHP2198 29017986 PMC5933323

[7] Dalsgaard A Frimodt-Møller N Bruun B Høi L Larsen JL . Clinical manifestations and molecular epidemiology of Vibrio vulnificus infections in Denmark. Eur J Clin Microbiol Infect Dis. 1996;15(3):227-32. 10.1007/BF01591359 8740858

[8] Ruppert J Panzig B Guertler L Hinz P Schwesinger G Felix SB Two cases of severe sepsis due to Vibrio vulnificus wound infection acquired in the Baltic Sea. Eur J Clin Microbiol Infect Dis. 2004;23(12):912-5. 10.1007/s10096-004-1241-2 15599654

[9] Andersson Y Ekdahl K . Wound infections due to Vibrio cholerae in Sweden after swimming in the Baltic Sea, summer 2006. Euro Surveill. 2006;11(8):E060803.2. 10.2807/esw.11.31.03013-en 16966771

[10] Lukinmaa S Mattila K Lehtinen V Hakkinen M Koskela M Siitonen A . Territorial waters of the Baltic Sea as a source of infections caused by Vibrio cholerae non-O1, non-O139: report of 3 hospitalized cases. Diagn Microbiol Infect Dis. 2006;54(1):1-6. 10.1016/j.diagmicrobio.2005.06.020 16368474

[11] Baker-Austin C Trinanes JA Salmenlinna S Löfdahl M Siitonen A Taylor NG Heat wave-associated vibriosis, Sweden and Finland, 2014. Emerg Infect Dis. 2016;22(7):1216-20. 10.3201/eid2207.151996 27314874 PMC4918148

[12] Brehm TT Berneking L Sena Martins M Dupke S Jacob D Drechsel O Heatwave-associated Vibrio infections in Germany, 2018 and 2019. Euro Surveill. 2021;26(41). 10.2807/1560-7917.ES.2021.26.41.2002041 34651572 PMC8518310

[13] Vogel MM Zscheischler J Wartenburger R Dee D Seneviratne SI . Concurrent 2018 hot extremes across northern hemisphere due to human-induced climate change. Earths Futur. 2019;7(7):692-703. 10.1029/2019EF001189 31598535 PMC6774312

[14] Kueh MT Lin CY . The 2018 summer heatwaves over northwestern Europe and its extended-range prediction. Sci Rep. 2020;10(1):19283. 10.1038/s41598-020-76181-4 33159097 PMC7648626

[15] Eurostat. Statistical regions in the European Union and partner countries. NUTS and statistical regions. 2020 edition. Luxembourg: Publications Office of the European Union; 2020. Available from: https://ec.europa.eu/eurostat/documents/3859598/10967554/KS-GQ-20-092-EN-N.pdf/9d57ae79-3ee7-3c14-da3e-34726da385cf

[16] Yu G Smith DK Zhu H Guan Y Lam TTY . ggtree: an R package for visualization and annotation of phylogenetic trees with their covariates and other associated data. Methods Ecol Evol. 2017;8(1):28-36. 10.1111/2041-210X.12628

[17] Nair GB Ramamurthy T Bhattacharya SK Dutta B Takeda Y Sack DA . Global dissemination of Vibrio parahaemolyticus serotype O3:K6 and its serovariants. Clin Microbiol Rev. 2007;20(1):39-48. 10.1128/CMR.00025-06 17223622 PMC1797631

[18] Baker-Austin C Trinanes J Gonzalez-Escalona N Martinez-Urtaza J . Non-cholera Vibrios: the microbial barometer of climate change. Trends Microbiol. 2017;25(1):76-84. 10.1016/j.tim.2016.09.008 27843109

[19] Romanello M McGushin A Di Napoli C Drummond P Hughes N Jamart L The 2021 report of the Lancet Countdown on health and climate change: code red for a healthy future. Lancet. 2021;398(10311):1619-62. 10.1016/S0140-6736(21)01787-6 34687662 PMC7616807

[20] Feingold MH Kumar ML . Otitis media associated with Vibrio alginolyticus in a child with pressure-equalizing tubes. Pediatr Infect Dis J. 2004;23(5):475-6. 10.1097/01.inf.0000126592.19378.30 15131479

[21] Kechker P Senderovich Y Ken-Dror S Laviad-Shitrit S Arakawa E Halpern M . Otitis media caused by V. cholerae O100: a case report and review of the literature. Front Microbiol. 2017;8:1619. 10.3389/fmicb.2017.01619 28894440 PMC5581382

[22] Baker-Austin C Oliver JD . Vibrio vulnificus: new insights into a deadly opportunistic pathogen. Environ Microbiol. 2018;20(2):423-30. 10.1111/1462-2920.13955 29027375

[23] Roux FL Wegner KM Baker-Austin C Vezzulli L Osorio CR Amaro C The emergence of Vibrio pathogens in Europe: ecology, evolution, and pathogenesis (Paris, 11-12th March 2015). Front Microbiol. 2015;6:830. 10.3389/fmicb.2015.00830 26322036 PMC4534830

[24] Eiler A Johansson M Bertilsson S . Environmental influences on Vibrio populations in northern temperate and boreal coastal waters (Baltic and Skagerrak Seas). Appl Environ Microbiol. 2006;72(9):6004-11. 10.1128/AEM.00917-06 16957222 PMC1563599

[25] Huehn S Eichhorn C Urmersbach S Breidenbach J Bechlars S Bier N Pathogenic vibrios in environmental, seafood and clinical sources in Germany. Int J Med Microbiol. 2014;304(7):843-50. 10.1016/j.ijmm.2014.07.010 25129553

[26] Böer SI Heinemeyer EA Luden K Erler R Gerdts G Janssen F Temporal and spatial distribution patterns of potentially pathogenic Vibrio spp. at recreational beaches of the German north sea. Microb Ecol. 2013;65(4):1052-67. 10.1007/s00248-013-0221-4 23563708

[27] Gyraite G Katarzyte M Schernewski G . First findings of potentially human pathogenic bacteria Vibrio in the south-eastern Baltic Sea coastal and transitional bathing waters. Mar Pollut Bull. 2019;149:110546. 10.1016/j.marpolbul.2019.110546 31543486

[28] European Centre for Disease Prevention and Control (ECDC). Vibrio map viewer. Stockholm: ECDC; 2018. Available from: https://www.ecdc.europa.eu/en/publications-data/vibrio-suitability-tool

[29] Chase E Harwood VJ . Comparison of the effects of environmental parameters on growth rates of Vibrio vulnificus biotypes I, II, and III by culture and quantitative PCR analysis. Appl Environ Microbiol. 2011;77(12):4200-7. 10.1128/AEM.00135-11 21515718 PMC3131657

[30] Laverty AL Primpke S Lorenz C Gerdts G Dobbs FC . Bacterial biofilms colonizing plastics in estuarine waters, with an emphasis on Vibrio spp. and their antibacterial resistance. PLoS One. 2020;15(8):e0237704. 10.1371/journal.pone.0237704 32804963 PMC7430737

[31] Lymperopoulou DS Dobbs FC . Bacterial diversity in ships’ ballast water, ballast-water exchange, and implications for ship-mediated dispersal of microorganisms. Environ Sci Technol. 2017;51(4):1962-72. 10.1021/acs.est.6b03108 28135081

[32] Fu S Hao J Yang Q Lan R Wang Y Ye S Long-distance transmission of pathogenic Vibrio species by migratory waterbirds: a potential threat to the public health. Sci Rep. 2019;9(1):16303. 10.1038/s41598-019-52791-5 31704994 PMC6841736

[33] Naseer U Blystad H Angeloff L Nygård K Vold L Macdonald E . Cluster of septicaemia and necrotizing fasciitis following exposure to high seawater temperatures in southeast Norway, June to August 2018. Int J Infect Dis. 2019;79(Supplement 1):28. 10.1016/j.ijid.2018.11.083

[34] Heng SP Letchumanan V Deng CY Ab Mutalib NS Khan TM Chuah LH Vibrio vulnificus: an environmental and clinical burden. Front Microbiol. 2017;8:997. 10.3389/fmicb.2017.00997 28620366 PMC5449762

[35] Trinanes J Martinez-Urtaza J . Future scenarios of risk of Vibrio infections in a warming planet: a global mapping study. Lancet Planet Health. 2021;5(7):e426-35. 10.1016/S2542-5196(21)00169-8 34245713

