# Supplementary material for: Epidemiological and microbiological investigation of a large increase in vibriosis, northern Europe, 2018
Source: Euro Surveill. 2022 Jul 14;27(28):2101088. doi: 10.2807/1560-7917.ES.2022.27.28.2101088 (PMC9284918; doi:10.2807/1560-7917.ES.2022.27.28.2101088)
Supplement: Supplement [file 21-01088_AMATO_Supplement.pdf]

SUPPLEMENTARY MATERIAL

This supplementary material is hosted by *Eurosurveillance* as supporting information alongside the article **Epidemiological and microbiological investigation of the large increase of vibriosis in northern Europe in 2018**, on behalf of the authors, who remain responsible for the accuracy and appropriateness of the content. The same standards for ethics, copyright, attributions and permissions as for the article apply. Supplements are not edited by *Eurosurveillance* and the journal is not responsible for the maintenance of any links or email addresses provided therein.

The present file contains supplementary information about the vibriosis surveillance system in the study countries, as well additional information of the vibriosis cases and related isolates of the project. Phylogenetic trees of *V. alginolyticus* and *V. parahaemolyticus* supporting our findings are also included.

Table S1. *Characteristics of national surveillance systems of vibriosis in study countries, 2018*

Country	Surveillance introduced	Data source	Reporting	Comprehensiveness	Design	Data reported by[1]	Data collection on all types of samples, regardless of infection severity[2]
Norway	No[3]	Laboratory database	Compulsory	Other[3, 4]	Passive	L	Yes
Sweden	Yes	National surveillance	Compulsory	Comprehensive	Passive	L, Ph, H	Yes
Denmark	No[5]	Laboratory database	Compulsory[5]	Other[5]	Passive	L	Yes
Finland	Yes	National surveillance	Compulsory	Comprehensive	Passive	L	Yes
Poland	Yes	National surveillance	Compulsory	Other[6]	Passive	L, Ph	Yes
Estonia	Yes	Laboratory database	Compulsory	Comprehensive	Passive	L	Yes

[1] *Data reported by: L – laboratories, Ph – physicians, H – hospitals.*

[2] *Severe infections (e.g. septicaemia, wound infections and necrotizing fasciitis) and non-severe infections (e.g. otitis, skin infections, gastroenteritis).*

[3] *Vibrio cholerae cases are routinely notified and isolates sent to the national reference laboratory at the national institute for the detection of cholera markers, wereas for non-cholerae Vibrio spp. this was the case for V. parahaemolyticus isolated from patients with gastrointestinal infections, only. Until 2019, other vibriosis were reported and isolates forwarded to the national institute in case of suspected outbreaks. Since 2019, vibriosis (irrespective of detected species) is a mandatory notifiable disease in Norway.*

[4] *Data collected through nationwide laboratories survey for the year 2018.*

[5] *Laboratory findings are automatically logged into the Danish Microbiology Database (MiBA), including infections caused by all* Vibrio *spp.*

[6] *Vibriosis cases were reported in the group "other bacterial foodborne infections" with only V. parahaemolyticus (ICD10 A05.3) specified as a separate vibriosis pathogen.*

Table S2. *Summary table of assembly and FASTQ statistics for Vibrio spp. isolates from study countries, 2014-2018 (Isolates are sorted by species).*

Isolate name[1]	Sequence type	Accession no.	Assembly statistics				FASTQ Statistics			
			Genome size	No. Contigs	Average size	N50	No Reads	Coverage	Mean Size read	SD Read length
NO_VA_18_19	96	ERS5890681	5421232	176	30802.45	315982	807569	236.415	146.374	14.7044
NO_VA_18_06	138	ERS5890687	5417902	190	28515.27	217049	948785	277.553	146.267	15.1111
NO_VA_18_01	180	ERS5890743	5407027	145	37289.84	276131	958833	283.302	147.733	11.0525
SE_VA_18_07	181	ERS5890679	5595925	107	52298.36	341854	3128519	883.332	141.174	21.1386
NO_VA_18_13	182	ERS5890690	5329433	158	33730.59	261597	826886	239.971	145.105	17.4573
NO_VA_18_22	183	ERS5890693	5374229	125	42993.83	301521	1215618	358.178	147.324	12.6567
NO_VA_18_02	184	ERS5890691	5262937	164	32091.08	236357	1009798	298.569	147.836	10.7947
SE_VA_18_05	185	ERS5890742	5294713	232	22822.04	335172	3332205	934.654	140.246	21.9543
NO_VA_18_18	186	ERS5890739	5161131	106	48689.92	474871	616064	182.035	147.74	11.4923
NO_VA_14_12	187	ERS5890686	5407567	164	32972.97	374289	555814	162.126	145.845	16.0321
NO_VA_18_24	188	ERS5890695	5156135	205	25151.88	243721	906604	263.139	145.124	17.2805
NO_VA_17_03	189	ERS5890688	5123679	145	35335.72	377119	668523	197.049	147.377	12.0064
SE_VA_18_01	189	ERS5890699	5209961	206	25291.07	306291	2342028	668.262	142.667	19.0984
SE_VA_18_03	189	ERS5890683	5245702	178	29470.24	306620	1991680	570.277	143.165	18.6206
NO_VA_14_08	190	ERS5890684	5344746	125	42757.97	285297	919872	268.354	145.865	15.9698
NO_VA_18_21	190	ERS5890698	5248406	113	46446.07	424596	859013	250.954	146.071	15.5318
NO_VA_14_09	191	ERS5890697	5126912	195	26291.86	184437	1063035	304.936	143.427	20.0946
NO_VA_14_10	192	ERS5890689	4856867	687	7069.68	12563	419714	109.1	129.97	31.0324
NO_VA_16_14	193	ERS5890741	5275994	185	28518.89	245908	1599715	450.432	140.785	23.0207
SE_VA_18_02	194	ERS5890678	5105536	150	34036.91	416418	2725118	786.1	144.232	16.6848
NO_VA_18_16	195	ERS5890685	5175692	190	27240.48	218225	381787	111.068	145.458	15.9461
NO_VA_18_17	196	ERS5890696	5199821	124	41934.04	486539	1030903	302.282	146.61	14.3043
NO_VA_15_20	197	ERS5890737	5221210	112	46617.95	461113	697234	204.871	146.917	13.571
NO_VA_18_11	198	ERS5890677	5371427	247	21746.67	262452	770781	224.259	145.475	16.5306
SE_VA_18_06	199	ERS5890740	5092698	148	34410.12	291124	2967297	846.333	142.61	19.1673
NO_VA_18_15	200	ERS5890682	5276788	213	24773.65	316620	2126896	594.344	139.721	24.7359
NO_VA_18_05	201	ERS5890694	5342558	191	27971.51	197410	2610356	716.865	137.312	27.1104
NO_VA_18_07	202	ERS5890680	5268605	123	42834.19	267069	1261658	363.711	144.14	19.021
NO_VA_18_23	202	ERS5890692	5204394	124	41970.92	368527	823506	241.841	146.836	14.0369
SE_VA_18_04	203	ERS5890738	5075931	187	27144.02	392950	823252	245.081	148.849	7.12042
NO_VA_14_04	204	ERS5890676	5179946	165	31393.61	446902	1318903	380.827	144.373	17.7441
SE_VC_18_18	8	ERS5890780	4085592	165	24761.16	177775	2515588	707.471	140.618	21.6816
SE_VC_18_19	8	ERS5890762	3999033	122	32778.96	247924	2724814	769.103	141.129	21.5285
NO_VC_17_02	86	ERS5890648	3994385	228	17519.23	86125	1356159	380.053	140.121	24.0678
FI_VC_18_04	88	ERS5890649	4123998	410	10058.53	148081	1197898	351.059	146.531	14.0905
PL_VC_16_02	88	ERS5890660	3992736	139	28724.72	155401	1091402	317.057	145.252	17.2512
SE_VC_18_05	88	ERS5890663	3966014	103	38504.99	155419	2563988	717.34	139.888	22.6501
PL_VC_18_01	303	ERS5890665	3987824	158	25239.39	246279	903757	264.063	146.092	15.5191
FI_VC_18_25	331	ERS5890668	4060283	228	17808.26	253959	2069404	606.635	146.573	14.0851
FI_VC_18_10	438	ERS5890645	4014291	281	14285.73	249848	2111542	619.075	146.593	14.0151
FI_VC_18_13	438	ERS5890640	4142630	312	13277.66	211126	1681522	492.875	146.556	14.0625
SE_VC_18_09	438	ERS5890744	3952491	144	27447.85	249848	1361757	398.922	146.473	12.8524
SE_VC_18_24	438	ERS5890703	3964416	199	19921.69	210781	3484277	990.524	142.142	19.7046
SE_VC_18_27	444	ERS5890734	3969652	204	19459.08	302560	2240015	636.282	142.026	20.197
DK_VC_18_02	452	ERS5890673	4065772	132	30801.3	128504	1368935	402.871	147.148	12.8087
SE_VC_18_22	464	ERS5890707	3946328	93	42433.63	271865	1410684	415.327	147.208	11.5934
SE_VC_18_43	464	ERS5890630	3948644	71	55614.7	572877	2958774	837.746	141.57	20.7317
SE_VC_18_23	470	ERS5890735	4023206	120	33526.72	217285	2355634	666.511	141.472	20.9195
FI_VC_18_34	488	ERS5890652	4460675	660	6758.6	210058	1520824	445.637	146.512	14.2519
FI_VC_18_33	492	ERS5890639	4120663	466	8842.62	237533	1778598	520.152	146.225	14.7686
SE_VC_18_42	497	ERS5890709	4044664	220	18384.84	184342	993932	295.96	148.884	7.38207
FI_VC_18_29	500	ERS5890643	4048074	317	12769.95	137653	1958806	574.459	146.635	13.9492
FI_VC_18_09	503	ERS5890662	4120619	322	12796.95	345276	2218474	645.657	145.518	16.1659
NO_VC_15_06	524	ERS5890755	4093649	191	21432.72	185142	563896	166.854	147.947	10.7875
NO_VC_16_01	557	ERS5890774	3925971	193	20341.82	146958	1224335	334.402	136.565	27.5007
DK_VC_18_03	558	ERS5890672	3988972	234	17046.89	141847	1514790	424.313	140.057	23.8934
DK_VC_18_04	697	ERS5890670	3884611	346	11227.2	111596	672384	196.088	145.815	15.5662

SE_VC_18_38	710	ERS5890765	4053433	254	15958.4	165378	2696100	774.056	143.551	17.9423
SE_VC_18_28	811	ERS5890764	4324690	289	14964.33	181503	2082543	593.734	142.55	19.347
FI_VC_18_15	967	ERS5890772	4254710	253	16817.04	150709	2078129	608.467	146.398	14.3373
NO_VC_18_05	988	ERS5890778	3951913	229	17257.26	152892	688787	200.279	145.385	16.69
FI_VC_18_16	1011	ERS5890627	4092488	209	19581.28	238852	1056143	308.994	146.284	14.5634
SE_VC_18_25	1035	ERS5890783	4048058	141	28709.63	217348	2578716	733.379	142.199	19.839
SE_VC_18_36	1079	ERS5890708	4062026	276	14717.49	256663	2256469	649.969	144.023	17.2764
PL_VC_15_03	1122	ERS5890769	4128666	123	33566.39	235089	700622	207.432	148.034	10.5464
SE_VC_18_35	1305	ERS5890752	4073088	270	15085.51	142229	1487203	432.762	145.495	15.1259
SE_VC_18_04	1306	ERS5890773	4013120	138	29080.58	270481	2329456	651.594	139.86	23.1752
FI_VC_18_08	1307	ERS5890756	4184733	349	11990.64	248241	2044865	598.571	146.36	14.5095
SE_VC_18_17	1308	ERS5890747	4051967	345	11744.83	311295	2118976	603.946	142.509	19.3805
FI_VC_18_37	1321	ERS5890664	5496910	3445	1595.62	3884	1769208	517.704	146.309	14.5189
FI_VC_18_14	1324	ERS5890651	4083111	380	10745.03	253365	1711347	501.994	146.666	13.8321
SE_VC_18_20	1324	ERS5890701	4025728	163	24697.72	203908	2562805	727.303	141.896	20.2302
FI_VC_18_36	1326	ERS5890736	5875531	5050	1163.47	2059	2834886	830.26	146.436	14.2883
FI_VC_18_39	1327	ERS5890777	6179601	4604	1342.22	2523	1921632	563.465	146.611	13.9655
FI_VC_18_03	1328	ERS5890771	4209040	336	12526.9	128998	944340	275.039	145.625	15.7838
FI_VC_18_19	1330	ERS5890657	4398920	556	7911.73	170863	1489878	435.609	146.189	14.8914
FI_VC_18_28	1330	ERS5890647	4093907	279	14673.5	181377	2067901	605.784	146.473	14.1882
FI_VC_18_35	1332	ERS5890656	4073160	335	12158.69	307485	2254360	661.036	146.613	13.9506
FI_VC_18_26	1335	ERS5890674	4057346	290	13990.85	198903	1605322	470.562	146.563	14.0913
NO_VC_18_10	1337	ERS5890761	4201468	400	10503.67	179016	1536627	437.171	142.25	21.4665
SE_VC_18_10	1341	ERS5890776	3972658	153	25965.08	240903	2670166	765.723	143.385	18.1254
SE_VC_18_21	1341	ERS5890750	3972808	154	25797.45	240771	2664167	758.575	142.366	19.6193
SE_VC_18_08	1343	ERS5890775	4579021	464	9868.58	55554	2399122	683.934	142.538	19.4236
FI_VC_18_31	1344	ERS5890650	4060116	411	9878.63	200339	2338586	684.911	146.437	14.3523
FI_VC_18_38	1365	ERS5890784	6036664	5200	1160.9	2041	2654973	778.285	146.571	14.0229
SE_VC_18_33	1368	ERS5890706	4032620	152	26530.39	334555	2184891	624.574	142.93	18.9731
FI_VC_18_23	1370	ERS5890641	4264238	535	7970.54	259073	1847148	539.58	146.058	15.0553
FI_VC_18_05	1376	ERS5890655	4257789	435	9788.02	252415	1169773	341.342	145.901	15.3938
FI_VC_18_18	1377	ERS5890751	4240020	617	6871.99	202624	1413805	412.443	145.863	15.4104
NO_VC_17_03	1378	ERS5890754	3953247	142	27839.77	234977	606394	177.859	146.653	14.0592
FI_VC_18_06	1382	ERS5890646	4213066	503	8375.88	180126	1476545	432.998	146.625	13.9515
SE_VC_18_40	1383	ERS5890702	3993134	172	23215.9	185950	3204566	906.099	141.376	20.8627
FI_VC_18_24	1393	ERS5890635	4253855	429	9915.75	346686	1804724	528.006	146.284	14.6164
SE_VC_18_34	1402	ERS5890704	4091017	195	20979.57	190389	3289550	934.944	142.108	19.8586
SE_VC_18_11	1406	ERS5890745	4082959	230	17752	157007	2807358	805.358	143.437	18.033
SE_VC_18_13	1406	ERS5890766	4110006	252	16309.55	157007	2094804	598.724	142.907	19.0521
SE_VC_18_41	1406	ERS5890759	4094914	227	18039.27	157632	3005353	856.381	142.476	19.4825
FI_VC_18_30	1407	ERS5890661	4115955	391	10526.74	182989	2267493	664.326	146.489	14.2163
SE_VC_18_07	1410	ERS5890785	3997947	151	26476.47	200755	2355879	663.645	140.849	21.707
SE_VC_18_30	1410	ERS5890770	4048778	142	28512.52	200792	2657256	754.751	142.017	20.1819
FI_VC_18_22	1425	ERS5890667	4207202	422	9969.67	195320	1471245	431.388	146.606	13.9786
SE_VC_18_14	1430	ERS5890705	4084169	277	14744.29	338961	2361486	676.297	143.193	18.515
SE_VC_18_29	1430	ERS5890753	4121961	289	14262.84	339029	2538178	720.615	141.955	20.417
FI_VC_18_07	1439	ERS5890638	4097939	353	11608.89	246500	1485128	435.006	146.454	14.3185
SE_VC_18_02	1440	ERS5890760	4027128	151	26669.72	246593	1871523	531.819	142.082	20.1539
FI_VC_18_11	1441	ERS5890636	4589003	773	5936.61	92152	1367795	400.869	146.539	14.1278
DK_VC_18_01	1442	ERS5890758	4105070	232	17694.27	90271	2427758	627.209	129.175	30.5484
FI_VC_16_01	1443	ERS5890710	4093432	374	10945.01	134899	1781910	522.741	146.68	13.8599
SE_VC_18_12	1444	ERS5890763	4070653	190	21424.49	171342	2933153	840.835	143.333	18.1592
NO_VC_17_04	1445	ERS5890669	4020954	199	20205.8	123186	520956	152.706	146.564	14.3174
NO_VC_17_09	1446	ERS5890659	3957109	227	17432.2	158315	577436	169.657	146.906	13.5845
NO_VC_17_11	1447	ERS5890757	3996588	175	22837.65	160525	783253	221.242	141.233	22.9442
NO_VC_15_07	1448	ERS5890628	4002234	170	23542.55	317992	431555	127.458	147.672	11.6002
FI_VC_18_17	1449	ERS5890700	4193491	524	8002.85	148010	2253099	661.224	146.737	13.6388
SE_VC_18_16	1450	ERS5890749	3925286	150	26168.57	199683	2192903	613.279	139.833	22.8064
SE_VC_18_39	1450	ERS5890782	3939055	172	22901.48	277126	1880124	538.351	143.169	18.6115
FI_VC_18_12	1451	ERS5890658	4199534	428	9812	131721	1859128	544.729	146.501	14.1309
SE_VC_18_03	1451	ERS5890746	4051940	184	22021.41	141073	1795077	511.278	142.411	19.9432
SE_VC_18_32	1451	ERS5890767	4043476	208	19439.79	132959	2272298	654.215	143.954	17.2797
SE_VC_18_26	1452	ERS5890629	4056334	150	27042.23	247076	3166412	897.225	141.678	20.5119
FI_VC_18_02	1453	ERS5890671	4295099	551	7795.1	258752	1745630	510.497	146.222	14.862

SE_VC_18_31	1453	ERS5890748	4083125	275	14847.73	259159	2191611	622.222	141.955	20.5207
SE_VC_18_37	1454	ERS5890786	4006908	173	23161.32	289893	1748603	498.275	142.478	19.6693
FI_VC_18_21	1455	ERS5890642	4217506	359	11747.93	299665	2016412	589.018	146.056	15.0806
FI_VC_18_20	1456	ERS5890666	4398960	503	8745.45	157740	1612774	473.043	146.655	13.8423
NO_VC_18_08	1457	ERS5890779	3922880	115	34112	248195	827482	243.432	147.092	13.1727
FI_VC_18_27	1458	ERS5890637	4096933	320	12802.92	276778	1773560	517.5	145.893	15.3816
FI_VC_18_32	1459	ERS5890654	4211988	350	12034.25	261943	2083644	611.023	146.624	13.9168
FI_VC_18_40	1460	ERS5890653	4253466	438	9711.11	514583	1636199	480.087	146.708	13.7508
SE_VC_18_01	1461	ERS5890781	4041774	224	18043.63	186142	2440169	692.796	141.957	20.193
SE_VC_18_06	1462	ERS5890768	4013885	230	17451.67	339329	2498845	686.51	137.365	25.9253
NO_VP_14_01	3	ERS5890798	5076372	127	39971.43	312643	1367888	398.367	145.614	15.5745
NO_VP_18_11	77	ERS5890719	4969676	94	52868.89	616547	614413	182.127	148.212	10.1077
NO_VP_18_12	479	ERS5890794	5358645	111	48276.08	515147	1778877	516.371	145.139	16.8569
NO_VP_18_05	483	ERS5890714	5340986	117	45649.45	361944	1000709	292.763	146.278	15.1041
NO_VP_18_08	766	ERS5890716	5051489	145	34837.86	331288	1562655	437.12	139.864	24.4253
NO_VP_18_10	767	ERS5890796	5162189	109	47359.53	327574	709200	208.573	147.048	13.3597
SE_VP_18_04	1081	ERS5890715	5086308	121	42035.6	455774	2697355	768.395	142.435	19.5158
SE_VP_18_08	1346	ERS5890792	5121318	118	43401	490626	2023103	577.674	142.769	19.1404
NO_VP_18_02	1372	ERS5890795	5026566	151	33288.52	193022	1780020	505.598	142.02	21.8689
SE_VP_18_06	1815	ERS5890791	5117517	75	68233.56	518931	3144612	896.21	142.499	19.2932
NO_VP_17_07	2240	ERS5890793	5266622	121	43525.8	469834	769824	222.389	144.442	18.5066
NO_VP_18_09	2414	ERS5890720	5289803	133	39772.95	390310	921920	269.202	146.001	15.8292
SE_VP_18_01	2415	ERS5890711	5019031	137	36635.26	329338	3343826	950.288	142.096	19.7324
SE_VP_18_05	2416	ERS5890713	5083570	158	32174.49	472036	2206774	632.948	143.41	18.2185
NO_VP_18_03	2417	ERS5890722	5164453	116	44521.15	539449	752819	220.308	146.322	14.9306
SE_VP_18_02	2418	ERS5890718	4926869	145	33978.41	326466	2821288	797.206	141.284	20.9407
NO_VP_16_06	2419	ERS5890797	5005092	152	32928.24	218250	2223756	585.281	131.597	30.951
SE_VP_18_07	2420	ERS5890721	5186572	118	43954	478064	2694201	762.377	141.485	20.781
NO_VP_14_04	2421	ERS5890717	5031640	110	45742.18	388862	711634	208.409	146.43	14.7628
SE_VP_18_03	2423	ERS5890712	5165336	73	70758.03	535832	2773380	784.773	141.483	20.8319
DK_VV_18_03	112	ERS5890803	4945660	302	16376.36	46709	1508698	398.522	132.075	29.4754
SE_VV_18_08	112	ERS5890724	5005813	313	15993.01	70053	2536020	725.399	143.019	18.5646
SE_VV_18_01	118	ERS5890631	5185854	352	14732.54	89585	2824385	807.726	142.991	18.629
SE_VV_18_07	128	ERS5890634	4828044	132	36576.09	192800	2513279	710.648	141.379	20.8896
NO_VV_18_08	143	ERS5890809	4984941	139	35862.88	220890	1090328	320.611	147.025	12.6556
SE_VV_18_05	226	ERS5890731	5047035	285	17708.89	120897	2592245	739.257	142.59	19.3631
FI_VV_18_01	253	ERS5890802	5104589	500	10209.18	205757	2029390	594.384	146.444	14.1642
DK_VV_18_01	531	ERS5890799	4953816	398	12446.77	145719	2215490	649.441	146.568	13.5376
SE_VV_18_02	531	ERS5890726	4966619	312	15918.65	131423	1987179	577.929	145.414	15.2668
SE_VV_18_06	531	ERS5890729	5029697	258	19494.95	131598	2585462	714.496	138.176	25.4798
DK_VV_18_02	532	ERS5890675	4980500	175	28460	161862	1613570	420.739	130.375	30.6238
DK_VV_18_04	533	ERS5890804	4929738	170	28998.46	103294	2046465	537.787	131.394	30.1469
NO_VV_18_06	534	ERS5890806	4978003	165	30169.72	508086	795812	234.211	147.152	12.542
NO_VV_18_07	534	ERS5890807	4930202	151	32650.34	450798	884153	260.226	147.161	12.5734
NO_VV_18_09	534	ERS5890730	4930278	182	27089.44	380496	592833	174.728	147.367	12.2702
NO_VV_18_10	534	ERS5890732	4982123	167	29833.07	436724	918507	271.017	147.531	11.8148
NO_VV_18_11	534	ERS5890632	4971254	178	27928.39	450859	744905	220.527	148.024	10.3071
NO_VV_18_01	534	ERS5890728	4967651	320	15523.91	130767	1207502	312.315	129.323	32.0266
NO_VV_18_02	534	ERS5890733	4928925	227	21713.33	431213	1330526	377.171	141.738	21.9846
NO_VV_18_03	534	ERS5890723	4962429	203	24445.46	333215	1084928	307.814	141.859	22.1973
NO_VV_18_05	534	ERS5890725	4931669	165	29888.9	333502	1031903	300.464	145.587	16.4905
NO_VV_18_04	535	ERS5890800	4947916	169	29277.61	400596	380987	112.627	147.81	11.2296
SE_VV_18_03	536	ERS5890633	4913018	268	18332.16	160710	3452420	979.215	141.816	20.2831
SE_VV_18_04	537	ERS5890727	5025044	223	22533.83	226159	2287089	648.342	141.739	20.4438
SE_VV_18_09	538	ERS5890801	5005214	164	30519.6	305804	3538537	988.566	139.686	22.7628
SE_VV_18_11	538	ERS5890805	4989246	162	30797.81	456888	2592338	738.138	142.369	19.5335
SE_VV_18_10	539	ERS5890808	4886578	465	10508.77	147117	2435150	693.834	142.462	19.613

[1]*The first abbreviation represents the country (DK – Denmark, FI – Finland, NO – Norway, SE – Sweden) and the second abbreviation the Vibrio species. (VA – Vibrio alginolyticus, VC – non-toxigenic Vibrio cholerae, VP – Vibrio parahaemolyticus, VV – Vibrio vulnificus). The first number represents the isolation year and the second number denotes the isolate number.*

Table S3. *Summary of key characteristics of non-travel related vibriosis cases in the study countries, 2018.*

Characteristic		Countries				
	Denmark	Sweden	Norway	Finland	Poland and Estonia	Total
Reported cases, n [a]	170	147	92	30	6	445
Male-to-female ratio (cases)	1.5 (101/69)	2.2 (101/46)	1.0 (47/45)	4.0 (24/6)	2.0 (4/2)	1.6 (277/168)
Notification rate per 100,000	2.9	1.4	1.7	0.5	ND	ND
Age, years						
median	53	58	42	39	34	ND
range	2-93	1-101	3-91	3-87	10-56	1-101
Age group, n (%)						
0-4	2 (1.2%)	3 (2%)	2 (2.2%)	2 (6.7%)	0 (0%)	9 (2%)
5-14	34 (20%)	24 (16.3%)	24 (26.1%)	7 (23.3%)	2 (33.3%)	91 (20.4%)
15-24	20 (11.8%)	14 (9.4%)	10 (10.8%)	2 (6.7%)	1 (16.7%)	47 (10.6%)
25-44	20 (11.8%)	16 (10.9%)	13 (14.1%)	5 (16.7%)	0 (0%)	54 (12.1%)
45-64	28 (16.4%)	31 (21.1%)	18 (19.6%)	4 (13.3%)	2 (33.3%)	83 (18.7%)
65-79	49 (28.8%)	35 (23.8%)	18 (19.6%)	6 (20%)	1 (16.7%)	109 (24.5%)
80+	17 (10%)	24 (16.3%)	7 (7.6%)	4 (13.3%)	0 (0%)	52 (11.7%)
Season, n (%)						
summer	134 (78.8%)	109 (74.2%)	57 (61.9%)	22 (73.4%)	4 (66.7%)	326 (73.3%)
autumn	29 (17.1%)	29 (19.7%)	31 (33.7%)	6 (20%)	1 (16.7%)	96 (21.6%)
winter	4 (2.3%)	5 (3.4%)	2 (2.2%)	1 (3.3%)	1 (16.6%)	13 (2.9%)
spring	3 (1.8%)	4 (2.7%)	2 (2.2%)	1 (3.3%)	0 (0%)	10 (2.2%)
Vibrio species, n (%)						
V. alginolyticus	70 (41.2%)	19 (12.9%)	63 (68.5%)	0 (0)	0 (0)	152 (34.2%)
Non-toxigenic *V. cholerae*	3 (1.8%)	64 (43.5%)	2 (2.2%)	26 (86.7%)	5 (83.3%)	100 (22.5%)
V. parahaemolyticus	55 (32.4%)	19 (12.9%)	12 (13.0%)	3 (10.0%)	0 (0)	89 (20.0%)
V. vulnificus	16 (9.4%)	19 (12.9%)	9 (9.8%)	1 (3.3%)	0 (0)	45 (10.1%)
Vibrio spp.	26 (15.3%)	26 (17.7%)	6 (6.5%)	0 (0)	1 (16.7%)	59 (13.3%)

[a] Data source: mandatory surveillance system (Sweden); nationwide surveying of public health microbiology laboratories (Norway); reporting of clinical sample analyses (Denmark, Finland, Poland, and Estonia). See material and methods for details on data acquisition. Note: ND – not determined.

Table S4. *Whole genome sequenced Vibrio isolates in the study countries, 2014-2018.*

Characteristics		Grand total	Total vibriosis		*Vibrio alginolyticus*		Non-toxigenic *Vibrio cholerae*		*Vibrio parahaemolyticus*		*Vibrio vulnificus*	
			nT	T	nT	T	nT	T	nT	T	nT	T
**Country**	Norway	58	35 (12)	3 (8)	16 (7)	0 (1)	2 (2)	1 (6)	6 (3)	2 (1)	11	0
	Denmark	8	6	2	0	0	2	2	0	0	4	0
	Sweden	68	66	2	6	1	41	1	8	0	11	0
	Finland	28	27	0 (1)	0	0	26	0 (1)	0	0	1	0
	Poland	3	1 (2)	0	0	0	1 (2)	0	0	0	0	0
**Sample type**	***Clinical***											
	Blood	46	43 (3)	0	1 (1)	0	18 (1)	0	4 (1)	0	20	0
	Faeces	20	7 (2)	4 (7)	0 (1)	0	6	3 (7)	1 (1)	1	0	0
	Ear-related	47	37 (6)	3 (1)	14 (3)	1 (1)	23 (2)	1	0 (1)	1	0	0
	Wound-related	34	32 (1)	0 (1)	5 (1)	0	14	0	8	0 (1)	5	0
	Other	18	16 (2)	0	2 (1)	0	11 (1)	0	1	0	2	0
	Environmental	13	13		0		13		0		0	
**Year**	2018	142	135	7	22	1	72	4	14	2	27	0
	2014-2017	23	14	9	7	1	4	7	3	1	0	0

nT – non-travel related clinical isolates or travel status unknown; T – travel related clinical isolates; All environmental were seawater isolates. Numbers indicate isolates of 2018 and in parenthesis isolates collected during 2014-2017.

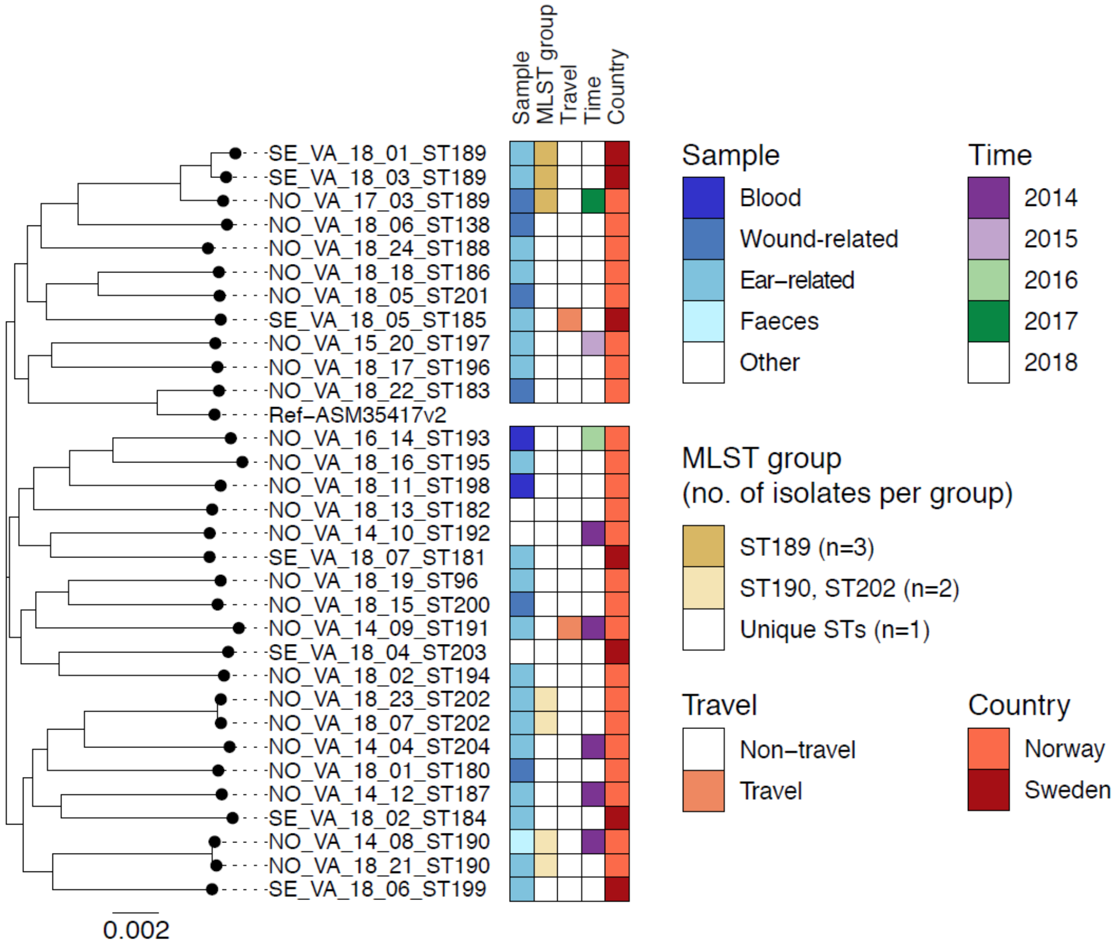

Figure S1. *SNP based phylogeny of 31 V. alginolyticus genomes from study countries.* The *V. alginolyticus* ASM35417v2 sequence was used as reference. The scale bar indicates the number of substitutions per site.

Note: VA, *V. alginolyticus*, NO – Norway, SE – Sweden, MLST – multilocus sequence typing, ST – sequence type. The first number represents the isolation year and the second number denotes the isolate number.

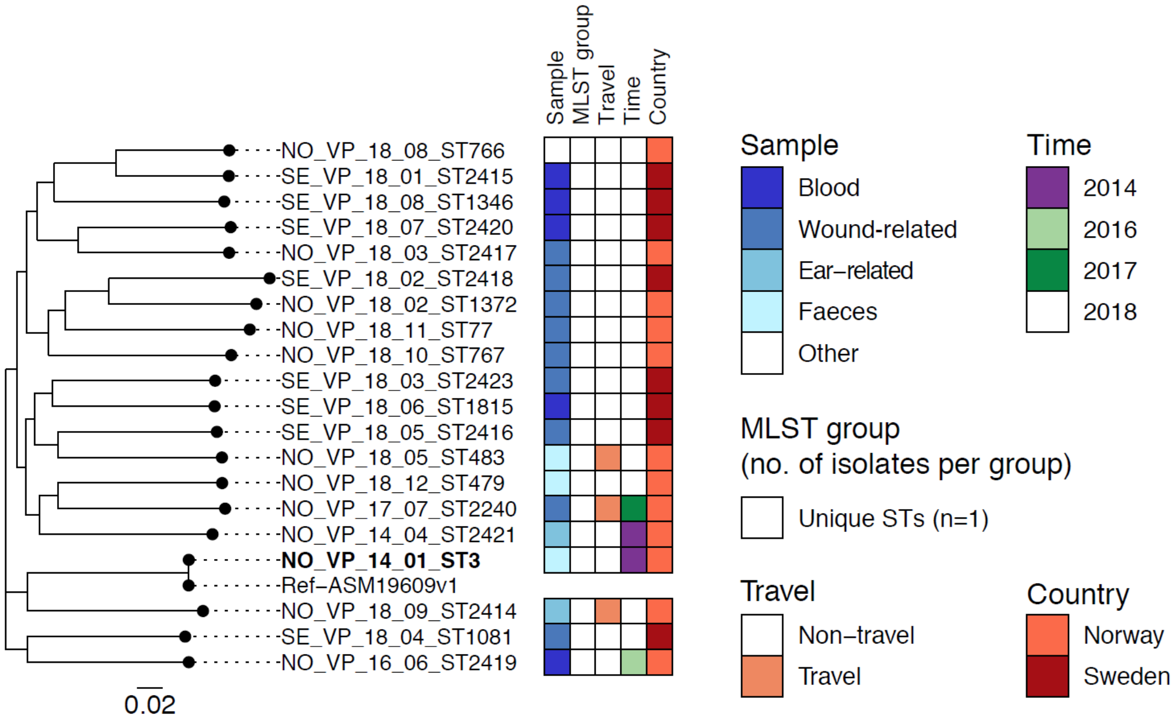

Figure S2. *SNP based phylogeny of 20 V. parahaemolyticus genomes from study countries.* The *V. parahaemolyticus* ASM19609v1 sequence was used as reference. The scale bar indicates the number of substitutions per site.

Note: VP – *V. parahaemolyticus*, NO – Norway, SE – Sweden, MLST – multilocus sequence typing, ST – sequence type. The first number represents the isolation year and the second number denotes the isolate number. Pandemic strain ST3 is bolded.
